# AIF, CK5/6, and CK20 in Bladder Urothelial Carcinoma: A Cross-Sectional Immunohistochemical Study of Grade and Stage Associations

**DOI:** 10.3390/jcm15124693

**Published:** 2026-06-17

**Authors:** Pavel Babal, Stefan Harsanyi, Sebastian Kern, Kristina Mikus Kuracinova, Lucia Krivosikova, Branislav Trebaticky, Stanislav Ziaran, Andrea Janegova, Pavol Janega

**Affiliations:** 1 Institute of Pathological Anatomy, Faculty of Medicine, Comenius University in Bratislava, 811 08 Bratislava, Slovakia; pavel.babal@fmed.uniba.sk (P.B.); kern9@uniba.sk (S.K.); kristina.kuracinova@fmed.uniba.sk (K.M.K.); lucia.krivosikova@fmed.uniba.sk (L.K.); andrea.janegova@fmed.uniba.sk (A.J.); pavol.janega@fmed.uniba.sk (P.J.); 2 Institute of Medical Biology, Genetics and Clinical Genetics, Faculty of Medicine, Comenius University in Bratislava, 811 08 Bratislava, Slovakia; 3 Department of Urology, Academician Ladislav Dérer Hospital, University Hospital Bratislava, 833 05 Bratislava, Slovakia; branotrebaticky@gmail.com; 4 Department of Urology, Ružinov Hospital, Faculty of Medicine, Comenius University and University Hospital Bratislava, 826 06 Bratislava, Slovakia; stanislav.ziaran@fmed.uniba.sk; 5 Institute of Normal and Pathological Physiology, Centre of Experimental Medicine, Slovak Academy of Sciences, 813 71 Bratislava, Slovakia

**Keywords:** bladder cancer, immunohistochemistry markers, cytokeratin 5/6, cytokeratin 20, apoptosis-inducing factor, stratification

## Abstract

**Background**: Most bladder cancer cases present as non-muscle-invasive bladder cancer (NMIBC), with the course of multiple recurrences leading to stage progression to muscle-invasive bladder cancer (MIBC) in 10–20% of cases, which is associated with higher morbidity and mortality. Accurate histopathologic classification of bladder cancer remains important for patient management. **Methods**: This retrospective–prospective observational cohort study was conducted on 244 transurethral resection specimens. Immunohistochemistry assessed CK5/6, CK20, and apoptosis-inducing factor (AIF) using three representations: intensity, percentage of positive cells, and multiplicative score. Discrimination between NMIBC (pTa/pT1) and MIBC (≥pT2), and between low-grade (LG) and high-grade (HG) tumors, was evaluated using ROC/AUC analysis and logistic regression. The main analysis focused on cross-sectional marker performance in primary/non-recurrent tumors. Recurrent tumors were analyzed only as an exploratory subgroup. Tumors were also categorized into basal, luminal, mixed/double-positive, and double-negative phenotypes using thresholds of 10% for CK5/6 and CK20. **Results**: For stage discrimination, all three markers showed modest separation. The best-performing representation was CK5/6 intensity (AUC 0.641; lower in MIBC). For grade discrimination, the AIF score showed the highest performance (AUC 0.729, higher in HG). Combining markers improved model performance (NMIBC vs. MIBC: AUC 0.784; strict LG vs. HG: AUC 0.778). Using the 10% cutoff in non-recurrent tumors, mixed/double-positive tumors had the lowest MIBC proportion (6.0%) and double-negative tumors the highest (46.7%). **Conclusions**: CK5/6, CK20, and AIF provide modest discrimination between stages, with lower CK5/6 and CK20, and higher AIF, in MIBC. The AIF score shows the highest separation between grades and may serve as a useful non-proliferation marker for grading, particularly when interpreted alongside CK5/6 and CK20 in a simple immunohistochemical panel.

## 1. Introduction

Worldwide, bladder cancer is the ninth most commonly diagnosed cancer and ranks thirteenth for cancer mortality. Bladder cancer continues to pose a significant global health challenge. However, temporal trends indicate a stabilizing or declining incidence in many high-income countries [1]. Urinary bladder cancer is primarily linked to environmental exposure to carcinogens (with cigarette smoking being the leading cause), including chronic industrial exposure to aromatic amines/dyes, chronic bladder irritation (e.g., infections or long-term catheter use), and following pelvic radiotherapy or chemotherapy [2,3]. When it occurs, approximately 75% of cases present with non-muscle-invasive bladder cancer (NMIBC), which has a typical clinical course of multiple recurrences, leading to stage progression to muscle-invasive bladder cancer in a subset (10–20%) of cases. The remaining 25% present as muscle-invasive bladder cancer (MIBC), which is associated with higher morbidity and mortality [4].

Bladder cancer is challenging and one of the most expensive cancer types to treat due to intensive follow-up regimens [1]. Accurate stratification of bladder cancer is crucial for effective health care management of patients with this disease. At present, none of the existing biomarkers can reliably predict tumor recurrence and/or stage progression. WHO grade and TNM stage remain among the most important prognostic factors for categorizing patients into low-, intermediate-, high-, and very high-risk groups [5]. NMIBC has unpredictable outcomes with a variable risk of recurrence and progression. Simple immunohistochemistry with two cytokeratins, CK5/6 and CK20, identifies the two major groups of urinary bladder carcinoma, the luminal and the basal types, and additionally includes two more subgroups—the mixed/double-positive and the double-negative types—which have shown clinical prognostic relevance [6,7]. Staining for these two cytokeratins has been shown to have prognostic and predictive value in the evaluation of bladder carcinoma [8,9], as well as serving as an additional helpful tool in the discrimination of in situ urothelial carcinoma from reactive atypia [10,11].

A six-group prognostic classification has been suggested for muscle-invasive tumors that correlates well with expression patterns detected in their NMIBC precursor lesions [5,12]. Many clinical and pathological prognostic factors have been identified but they remain insufficient, raising the need to investigate additional biomarkers [1,4,13,14]. Identifying novel biomarkers and molecular mechanisms is crucial for enhancing early detection and the development of targeted therapies. Apoptosis-inducing factor (AIF) is a bifunctional flavoprotein that exhibits distinct actions depending on its cellular compartment. It can activate apoptosis in a caspase-independent manner. Also, AIF plays an important role in maintaining mitochondrial morphology and energy metabolism [15,16].

The aim of this study was to evaluate the cross-sectional associations of CK5/6, CK20, and AIF expression with tumor stage and grade in bladder urothelial carcinoma and to compare the discriminatory performance of different immunohistochemical scoring approaches. The main analysis focused on primary/non-recurrent tumors, while recurrent tumors were analyzed separately as an exploratory subgroup rather than as a longitudinal follow-up cohort [12,14,17].

## 2. Materials and Methods

### 2.1. Study Design

This retrospective–prospective observational cohort study was conducted at the University Hospital in Bratislava. The study was designed as a cross-sectional evaluation of IHC marker performance for pathological stage and grade discrimination, with emphasis on primary/non-recurrent tumors, rather than as a longitudinal follow-up or outcome-prediction study. Tissue samples from patients with urothelial carcinoma of the urinary bladder were obtained by transurethral resection (TURB) with complete macroscopic excision and collected between August 2022 and January 2025 at the Department of Urology of Comenius University and of University Hospital Bratislava. Cases collected before ethical approval were included retrospectively from archived TUR specimens and anonymized records, whereas cases collected after 8. August 2024 were included prospectively. Radical cystectomies and cases with non-neoplastic diagnoses or tumors at other sites were excluded. Only anonymized clinical and pathological data were used, with no additional interventions beyond routine care.

The study analyzed anonymized clinical and pathological data from patients with urothelial bladder cancer, which included demographic variables (age, sex), tumor characteristics (tumor type, count, stage, and grade), and recurrence status. Grading was evaluated using a two-tier system (low grade vs. high grade), and pathological stage was categorized as NMIBC (Ta–T1) and MIBC (≥T2). Expanded grades HG > LG and HG < LG were determined in the 5–50% interval of HG tumor tissue. The diagnosis was made by two independent pathologists in accordance with the WHO 2022 criteria [4]. Immunohistochemical (IHC) analysis included assessment of the expression levels of cytokeratins CK5/6 and CK20, as well as apoptosis-inducing factor (AIF).

The primary objective was to assess the association of these markers with tumor stage and grade and to compare the discriminatory performance of three immunohistochemical readouts: staining intensity, percentage of positive cells, and multiplicative score. Analyses were performed in the overall cohort and separately in primary and recurrent tumors to determine whether marker performance differed between these clinical settings.

### 2.2. Histology, Immunohistochemistry

Surgical specimens were fixed in formalin and routinely processed in paraffin; 4-μm sections were stained with hematoxylin and eosin and evaluated under light microscopy. Immunohistochemical staining was performed on deparaffinized 4 μm thick sections in a DAKO Autostainer after antigen retrieval in PTlink (DAKO, Glostrup, Denmark) according to the manufacturer’s instructions. We applied ready-to-use prediluted antibodies against cytokeratins 5/6 (CK5/6) (Agilent Technologies, Santa Clara, CA, USA), and antibodies diluted in Dako Real antibody diluent in the following ratios: CK20 1:50 (Agilent Technologies); apoptosis-inducing factor (AIF) (Santa Cruz Biotechnology, Dallas, TX, USA) 1:500.

Microscopic assessment and figure documentation were performed using an Eclipse Cί light microscope (Nikon, Tokyo, Japan). Cytokeratin and AIF expression were semiquantitatively assessed by cytoplasmic staining, with 4 levels of intensity: negative—0 (absence of staining), weak—1 (faint but clearly detectable staining), moderate—2 (easily detectable staining of intermediate intensity), and strong—3 (high staining intensity). The percentage of positive tumor cells was recorded as the proportion of tumor cells showing unequivocal specific staining and was expressed as a value from 0% to 100%. The staining parameters were statistically evaluated individually or as multiplicative scores.

Marker-specific sample sizes differed because some IHC stains or scoring components were not evaluable in all cases. Missing values resulted mainly from limited residual tumor tissue in TUR specimens, tissue loss or exhaustion on deeper sections, technical artifacts, or non-interpretable staining for a given marker. Therefore, intensity, percentage of positive cells, and multiplicative scores were analyzed only when the corresponding staining component was assessable. Complete-case analysis was applied for each marker representation and endpoint.

### 2.3. Statistical Analysis

Statistical analysis was performed using Microsoft Excel (Microsoft Corp., Redmond, WA, USA) and IBM SPSS Statistics version 29 (IBM Corp., Armonk, NY, USA). Continuous variables are reported as median and range, and categorical variables as number and percentage. Blank values were treated as missing. All inferential analyses were performed using complete cases for the variables required in each test.

For cross-sectional analyses, only one specimen per subject was included. Primary-tumor analyses were restricted to primary/non-recurrent tumors. Recurrent tumors included in the overall analysis represented independent subjects. Serial recurrent specimens from the same subject were excluded from the overall ROC and logistic regression analyses and were assessed separately in the curated longitudinal recurrence dataset.

For CK5/6, CK20, and AIF IHC readouts, discriminatory performance was assessed by ROC analysis with calculation of the area under the curve (AUC) and 95% confidence intervals. The main comparisons were NMIBC versus MIBC (defined as pTa/pT1 versus ≥pT2) and strict LG versus HG tumors. An expanded grade comparison, including intermediate grade groups, was also performed. Marker combinations were evaluated using multivariable logistic regression, and model performance was summarized by AUC.

For phenotype analysis, tumors were classified as basal, luminal, mixed/double-positive, or double-negative, using a 10% positivity cutoff for CK5/6 and CK20. This cutoff was selected as a pragmatic threshold for marker positivity, consistent with prior IHC scoring approaches in urothelial carcinoma, in which staining below 10% was considered negative. Associations between phenotype and clinicopathological variables were assessed using the χ^2^ test or Fisher’s exact test, as appropriate. The added value of AIF beyond phenotype was examined using logistic regression and compared using the likelihood ratio test. In the recurrent-only dataset, within-patient changes between consecutive tumors were assessed descriptively and by exact nonparametric directional testing. All tests were two-sided, and *p* < 0.05 was considered statistically significant.

## 3. Results

### 3.1. Distribution of Clinicopathological Variables

A total of 244 TURB specimens were included, corresponding to 244 unique biopsy IDs. In total, 190 (77.9%) were primary tumors, and 54 (22.1%) were recurrent tumors. The median age was 70 years (range 39–92). Sex was available for all cases, with 178 males (73.0%) and 66 females (27.0%). The most frequent histological category was non-invasive urothelial carcinoma LG (110/244, 45.1%), followed by invasive urothelial carcinoma (84/244, 34.4%) and non-invasive urothelial carcinoma HG (35/244, 14.3%). Stage pTa was the most frequent stage (151/244, 61.9%), followed by pT1 and ≥pT2. Lymphovascular invasion (LVI) was absent in most cases (196/244; 80.3%). The distribution of clinicopathological variables is presented in Table 1.

### 3.2. Comparison Between Marker Intensity, Percentage, and Multiplicative Score

**Figure 1:**
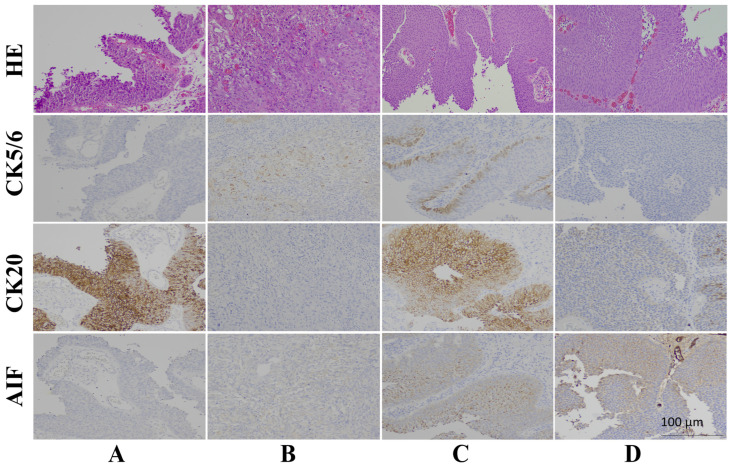
Immunohistochemical staining of TURB specimens of bladder urothelial carcinoma using antibodies against CK5/6, CK20, and AIF. Based on cytokeratin expression, luminal (**A**), basal (**B**), double-positive (**C**), and double-negative (**D**) phenotypes are identified. HE = hematoxylin and eosin; immunoperoxidase technique with hematoxylin counterstaining; original magnification ×100.

To determine which immunohistochemical parameter most accurately reflects tumor aggressiveness, we systematically evaluated CK5/6, CK20, and AIF using three different representations: intensity, percentage of positive cells, and combined multiplicative score. Representative images of the evaluated immunohistochemical staining, arranged into the basal, luminal, double-positive, and double-negative groups, are presented in Figure 1.

Because different studies use different scoring approaches, we aimed to identify which metric provides the best discrimination between clinically relevant groups, namely NMIBC vs. MIBC and LG vs. HG tumors. By comparing ROC-derived AUC values across representations, we assessed not only whether a marker is informative but also which quantification method yields the highest diagnostic performance.

Table 2 shows that all three markers provided modest separation between NMIBC and MIBC in non-recurrent tumors. CK20 tended to be lower in MIBC across all three representations, and CK5/6 also tended to be lower in MIBC across all three representations, whereas AIF tended to be higher in MIBC. The best-performing single-marker representations for stage discrimination were AIF score (AUC 0.655), CK5/6 intensity (AUC 0.650), and CK20% (AUC 0.638), indicating that the most informative metric differed by marker.

Table 3 demonstrates stronger discrimination between tumor grades than between stages, driven mainly by AIF. AIF score showed the highest accuracy for strict HG vs. LG discrimination (AUC 0.727), with AIF% and AIF intensity also performing well. CK5/6 showed a consistent tendency toward lower expression in HG, with the best performance obtained for CK5/6 score (AUC 0.640) and CK5/6% (AUC 0.636). CK20 provided only modest discrimination, with the best performance obtained for CK20% (AUC 0.582), whereas CK20 intensity showed no useful separation. Thus, among the single markers, AIF contributed the most to grade separation, whereas CK5/6 remained directionally consistent across all three representations.

Because none of the individual markers reached a level sufficient for stand-alone clinical use, we also tested marker combinations. For NMIBC vs. MIBC in non-recurrent tumors, the best combination was CK5/6 intensity + CK20 intensity + AIF score, which improved discrimination to AUC 0.784. For strict LG vs. HG, the best-performing combination was CK5/6 intensity + CK20 score + AIF score, which yielded AUC 0.782.

In the expanded grade comparison, including LG > HG and HG > LG, CK5/6% remained the best CK5/6 representation (AUC 0.659), with lower values in HG-predominant tumors, whereas the AIF score showed the highest performance overall (AUC 0.701), with higher values in HG-predominant tumors. CK20 discrimination remained limited, with the best performance achieved by score (AUC 0.562), suggesting that CK20 contributes less to grade separation than CK5/6 or AIF in this non-recurrent cohort.

For LVI in non-recurrent tumors (151 LVI−, 39 LVI+), CK5/6 intensity was lower, and AIF score was higher in LVI-positive tumors (*p* = 0.0066 and *p* = 0.0119, respectively). AIF% was also nominally higher (*p* = 0.0424), whereas CK20 and the remaining CK5/6 representations did not differ significantly. After Bonferroni correction, none of these associations remained statistically significant.

### 3.3. Subgroup Analysis of Recurrent-Only Tumors

The recurrent-only subgroup included 33 subjects and 87 biopsies, corresponding to 54 recurrent tumors. This analysis was performed as an exploratory within-patient longitudinal assessment in a curated serial-biopsy dataset. Because recurrent biopsies were obtained opportunistically rather than through a predefined follow-up protocol, these results were interpreted descriptively.

For recurrence-onset analysis, the biopsy immediately preceding the first documented recurrence was compared with the first recurrent biopsy within each subject, yielding 31 evaluable first-recurrence transitions. No marker showed a statistically significant directional change at the onset of recurrence. CK5/6 score decreased in 16/28 non-tied transitions, whereas CK20 score and AIF score changed bidirectionally without a consistent pattern.

Eight TNM upstaging transitions were identified. CK5/6 score decreased in six out of seven non-tied pairs, suggesting a possible association between loss of CK5/6 and stage progression; however, this trend did not reach statistical significance. CK20 and AIF did not show consistent directional changes. Epirubicin status before the first recurrence was available in 10/21, and BCG in only 2/31 evaluable transitions. After epirubicin, the following recurrence was usually unchanged in stage and grade, while marker scores, especially CK5/6, changed more often, with a weak tendency toward lower CK5/6 in the next recurrence.

### 3.4. Subgroup Analysis of Markers Between Primary and Recurrent Cases

Restricting the analysis to primary vs. recurrent tumors yielded more informative results. The results are shown in Table 4.

For stage discrimination, the best single-marker performance in the whole cohort was observed for CK5/6 intensity (AUC 0.641), followed by CK20 intensity (AUC 0.637) and AIF score (AUC 0.632). In primary tumors, the AIF score was the best single marker (AUC 0.655), followed by CK5/6 intensity (AUC 0.650) and CK20% (AUC 0.638). In recurrent tumors, the highest stage-related AUC was observed for CK20 intensity (AUC 0.671), followed by the CK20 score (AUC 0.665) and the AIF score (AUC 0.650), although these estimates should be interpreted with caution given the smaller sample size.

For strict grade discrimination, the AIF score was the strongest individual marker in all three cohorts, with AUCs of 0.729 in the whole cohort, 0.727 in primary tumors, and 0.754 in recurrent tumors. In the whole cohort and in primary tumors, AIF% was the second-best marker, whereas CK5/6 score and CK5/6% showed the most consistent inverse association with high-grade tumors. Overall, the discrimination pattern was broadly preserved across cohorts, with AIF score remaining the most robust single marker for grade and CK5/6/CK20-based measures contributing most to stage discrimination.

### 3.5. Subgroup Analysis of AIF Contribution to Primary Tumors

In primary tumors only, using a 10% positivity cutoff for CK5/6 and CK20, 187/190 tumors were classifiable into four pragmatic IHC phenotypes: mixed/double-positive (67/187, 35.8%), luminal (66/187, 35.3%), basal (39/187, 20.9%), and double-negative (15/187, 8.0%) [18].

Mixed/double-positive tumors showed the lowest proportion of MIBC (4/67, 6.0%), whereas double-negative tumors showed the highest proportion (7/15, 46.7%), consistent with a more dedifferentiated phenotype. Luminal tumors were the most enriched for strict HG disease (33/47, 70.2%), whereas lower proportions were observed in basal (9/26, 34.6%), mixed (20/48, 41.7%), and double-negative tumors (6/11, 54.5%).

AIF tended to be higher in luminal and double-negative phenotypes than in basal tumors. Median AIF% values were 70% in luminal tumors, 70% in double-negative tumors, 50% in basal tumors, and 50% in mixed tumors. The median recalculated AIF score values were 90, 70, 55, and 75, respectively.

When AIF% was added to the phenotype-based model in cases with complete data (n = 165), model fit improved slightly, but did not reach formal statistical significance (LR *p* = 0.057). Thus, in primary tumors, the basal–luminal phenotype itself remains informative, and the independent added predictive value of AIF beyond phenotype is only suggestive.

## 4. Discussion

From the results of numerous studies, it is clear that bladder cancer is a heterogeneous disease not only in terms of clinical and pathological characteristics but also in terms of molecular alterations [19,20,21]. Recently, great progress has been made in the molecular classification of urothelial bladder cancer [19]. The introduction of the two major categories, basal and luminal types, established the basic prognostic differentiation of bladder cancer [21,22]. However, basal/luminal subtyping and commonly used immunohistochemical markers are not yet incorporated into routine guideline-based grading, staging, or treatment decision-making for NMIBC/MIBC. Grading of urinary bladder cancer was unified into the more reproducible LG and HG categories. Although a four-tier grading system for NMIBC has been suggested to provide enhanced prognostic value [4,23]. With advances in next-generation sequencing and bioinformatics, additional distinct molecular subtypes of BC have been revealed [5,6,7]. The expression of cytokeratins 5 and 20 has been associated with the basal and luminal molecular subtypes of the tumor. Recently, CK20 expression was shown to positively correlate with HER2 overexpression, whereas CK5/6 expression correlates with PD-L1 expression. These associations gain significant therapeutic implications [12,24].

In the present study, we used CK5/6 as a basal marker and CK20 as a luminal marker and evaluated them together with AIF using three representations: intensity, percentage of positive cells, and multiplicative score. This approach showed that the clinicopathological informativeness of these markers depends not only on the marker itself but also on how expression is quantified. Across the primary-diagnosed cohort, discrimination between NMIBC and MIBC was modest, with CK5/6 and CK20 generally lower in MIBC and AIF generally higher. Grade discrimination was more pronounced and was driven mainly by AIF, particularly when assessed as a multiplicative score. However, the observed AUC values do not support stand-alone clinical classification. These markers should therefore be interpreted as supportive adjuncts to morphology, WHO grade, and TNM stage, rather than as replacements for established diagnostic criteria. CK20 contributed more clearly to stage than to grade discrimination, whereas AIF added more information for grade-oriented interpretation.

Our use of CK5/6 and CK20 to approximate basal-luminal differentiation is supported by previous studies showing that simplified IHC-based panels can provide clinically meaningful subtype information in routine practice [25]. Similar IHC-oriented approaches have also been applied in institutional bladder cancer cohorts and routine pathology settings [12,26]. As in other reports, we also identified mixed/double-positive and double-negative tumors in addition to the classical basal and luminal groups [26,27]. In the subgroup analysis restricted to primary-diagnosed tumors, mixed/double-positive and luminal phenotypes were the most frequent, accounting for 35.8% and 35.3% of classifiable tumors, followed by basal (20.9%) and double-negative (8.0%) phenotypes [28,29]. Importantly, the lowest proportion of MIBC was observed in the mixed/double-positive phenotype, whereas the highest proportion was found in the double-negative phenotype, consistent with a more dedifferentiated pattern [27]. Luminal tumors were the most enriched for strict HG disease. These findings suggest that, in TUR-derived material, the mixed and double-negative phenotypes may be more informative than a simple binary basal–luminal split.

A particularly relevant finding of the present study is the role of AIF. Higher AIF expression was associated with HG disease across all three readouts, but the strongest and most consistent signal was obtained with the multiplicative score. The AIF score also contributed to stage discrimination, although less strongly than to grade discrimination. In primary-diagnosed tumors, it became the best single marker for both stage and grade. The biological plausibility of this finding is supported by the known role of AIF as a mitochondrial flavoprotein involved in oxidative phosphorylation, redox regulation, and cellular stress responses [30]. Its broader relevance as a prognostic biomarker has also been reported in other tumor types, although the prognostic direction has not been entirely uniform across cancers [15,16]. Experimental work has further suggested that AIF can influence stress-response and signaling pathways relevant to tumor progression [31]. In our material, AIF showed predominantly granular cytoplasmic staining, consistent with mitochondrial localization and therefore with its metabolic rather than nuclear apoptotic function. Experimental data from bladder cancer models have further suggested that AIF-related pathways may contribute to treatment-induced cell death [32].

CK5/6 showed a stable inverse relationship with both stage and grade, suggesting progressive loss of basal differentiation in more aggressive tumors. CK20, in contrast, contributed more to stage discrimination than to grade separation. Previous studies have linked CK20 expression to tumor progression and poor prognosis in advanced bladder cancer, indicating that its role may vary depending on the biological context and disease subset under study [17]. Our findings suggest that, within a simplified IHC panel, CK20 may be more useful for stage-oriented discrimination, whereas AIF adds more information for grading.

In the cross-sectional comparison, no marker showed a significant association with recurrence status, although recurrent tumors tended to have higher AIF and lower CK5/6 values. In the curated serial-biopsy recurrence dataset, no marker demonstrated a significant directional change at recurrence onset. However, CK5/6 most often decreased in tumors that underwent TNM upstaging, suggesting that loss of CK5/6 may accompany progression in at least a subset of recurrent cases. Because the number of evaluable transitions was small, this observation should be regarded as hypothesis-generating. Similarly, the available data on BCG and epirubicin were too sparse to support robust treatment-stratified conclusions.

The present study has several limitations, including its retrospective design, reliance on TUR specimens only, single-institution cohort, and relatively small recurrent serial-biopsy subgroup, which may limit generalizability. The study was exploratory and not designed to assess clinical outcomes. Therefore, no conclusions can be drawn regarding recurrence-free survival, progression-free survival, disease-specific survival, treatment response, or overall survival. No formal a priori sample-size or power calculation was performed, as case inclusion was based on available TUR specimens collected during the study period. Although ROC analysis and logistic regression were applied, adjustment for potential confounders was limited by subgroup size, missing values, and the number of events in certain comparisons, particularly in comparisons involving recurrent tumors. Marker-specific missingness in IHC data may also have introduced selection bias, especially in AIF-based analyses, although missingness was mainly related to tissue availability or technical interpretability rather than to known clinicopathological characteristics. Finally, the immunophenotypic classification was intentionally simplified for routine diagnostic applicability and therefore does not capture the full complexity of contemporary molecular subtype systems. However, this pragmatic approach also represents a strength by reflecting real-world pathology practice.

## 5. Conclusions

All three markers showed modest discriminatory ability in primary-diagnosed tumors for NMIBC (pTa/pT1) versus MIBC (pT2+), with CK5/6 and CK20 generally lower in MIBC and AIF generally higher in MIBC. In contrast, grade discrimination was more pronounced than stage discrimination, and AIF score consistently showed the best performance for distinguishing HG from LG tumors. CK5/6 retained a stable inverse association with tumor aggressiveness, whereas CK20 contributed more to stage than to grade separation. Overall, these findings suggest that AIF, particularly when assessed as a multiplicative score, may serve as a supportive non-proliferation marker for bladder cancer grading, especially when interpreted alongside CK5/6 and CK20 within a simple immunohistochemical panel.

## Figures and Tables

**Table 1:** Distribution of clinicopathological variables in the study group.

Variable	Category	n (%)
Stage	pTa	151 (61.9)
	pT1	45 (18.4)
	≥pT2	48 (19.7)
Grade	LG	86 (35.2)
	LG > HG	45 (18.4)
	HG > LG	22 (9.0)
	HG	91 (37.3)
Recurrent tumor	No	190 (77.9)
	Yes	54 (22.1)
Lymphovascular invasion	No	196 (80.3)
	Yes	48 (19.7)

**Table 2:** Analysis of markers based on stage discrimination in primary tumors (NMIBC vs. MIBC).

Marker	Representation	n	AUC (95% CI)	Direction in MIBC
CK5/6	Intensity	190	0.650 (0.550–0.745)	Lower
CK5/6	%	189	0.602 (0.476–0.728)	Lower
CK5/6	Score	189	0.607 (0.481–0.731)	Lower
CK20	Intensity	188	0.627 (0.510–0.739)	Lower
CK20	%	188	0.638 (0.521–0.750)	Lower
CK20	Score	187	0.631 (0.507–0.740)	Lower
AIF	Intensity	169	0.603 (0.502–0.705)	Higher
AIF	%	168	0.604 (0.488–0.711)	Higher
AIF	Score	168	0.655 (0.535–0.767)	Higher

**Table 3:** Analysis of markers based on grade discrimination in primary tumors (strict LG vs. HG).

Marker	Representation	n	AUC (95% CI)	Direction in HG
CK5/6	Intensity	135	0.621 (0.531–0.707)	Lower
CK5/6	%	134	0.636 (0.539–0.725)	Lower
CK5/6	Score	134	0.640 (0.543–0.729)	Lower
CK20	Intensity	133	0.488 (0.388–0.583)	No clear pattern
CK20	%	133	0.582 (0.481–0.680)	Higher
CK20	Score	132	0.576 (0.478–0.676)	Higher
AIF	Intensity	118	0.627 (0.538–0.716)	Higher
AIF	%	118	0.678 (0.580–0.769)	Higher
AIF	Score	118	0.727 (0.630–0.817)	Higher

**Table 4:** Comparison of marker discriminatory performance.

Cohort	Endpoint	n	Best Marker	AUC	2nd Best Marker	AUC
All	NMIBC vs. MIBC	190	CK5/6 intensity	0.641	CK20 intensity	0.637
	LG vs. HG	177	AIF score	0.729	AIF%	0.654
Primary	NMIBC vs. MIBC	190	AIF score	0.655	CK5/6 intensity	0.650
	LG vs. HG	135	AIF score	0.727	AIF%	0.678
Recurrent	NMIBC vs. MIBC	54	CK20 intensity	0.671	CK20 score	0.665
	LG vs. HG	42	AIF score	0.754	AIF%	0.726

## Data Availability

The original contributions presented in this study are included in the Supplementary Material (Table S1: dataset.xlsx). Further inquiries can be directed to the corresponding author.

## Supplementary Materials

The following supporting information can be downloaded at: https://www.mdpi.com/article/10.3390/jcm15124693/s1, Table S1: dataset.xlsx.
